# Tamm plasmon modes on semi-infinite metallodielectric superlattices

**DOI:** 10.1038/s41598-017-03497-z

**Published:** 2017-06-16

**Authors:** Goran Isić, Slobodan Vuković, Zoran Jakšić, Milivoj Belić

**Affiliations:** 1 Institute of Physics Belgrade, Center for Solid State Physics and New Materials, University of Belgrade, Belgrade, 11080 Serbia; 2 Institute of Chemistry, Technology and Metallurgy, Center Of Microelectronic Technologies, University of Belgrade, Belgrade, 11000 Serbia; 3 Texas A&M University at Qatar, Doha, P.O. Box 23874, Qatar

## Abstract

We analyze the fundamental properties of optical waves referred to as Tamm plasmon modes (TPMs) which are tied to the interface of a semi-infinite two-phase metallodielectric superlattice with an arbitrary homogeneous capping medium. Such modes offer new ways of achieving high electromagnetic field localization and spontaneous emission enhancement in the vicinity of the interface in conjunction with absorption loss management, which is crucial for future applications. The homointerface, formed when the capping medium has the same permittivity as one of the superlattice constituents, is found to support a TPM whose dispersion overlaps the single-interface surface plasmon polariton (SPP) dispersion but which has a cut off at the topological transition point. In contrast, a heterointerface formed for an arbitrary capping medium, is found to support multiple TPMs whose origin can be traced by considering the interaction between a single-interface SPP and the homointerface TPM burried under the top layer of the superlattice. By carrying out a systematic comparison between TPMs and single-interface SPPs, we find that the deviations are most pronounced in the vicinity of the transition frequency for superlattices in which dielectric layers are thicker than metallic ones.

## Introduction

The development of nanofabrication techniques has recently enabled the experimental demonstration of various artificial materials consisting of subwavelength metallodielectric elements - metamaterials, designed to exhibit peculiar optical properties that are not present in conventional media^1^. Aimed at gaining control over light propagation, any artificial optical material must rely on a high radiation confinement and low losses in the structure composites^2^. Surface modes on metal-dielectric interfaces offer a high radiation confinement to the surface, but the presence of intrinsic dissipation in the metallic component imposes severe restrictions to their applications.

Among a variety of metamaterials that have been designed and fabricated so far, the so-called hyperbolic metamaterials (HMMs) have attracted a rapidly growing attention^3^, as high quality ultrathin metal films can be grown^4^ yielding metallodielectric superlattices that support electromagnetic modes with very high wavenumbers and large photonic density of states that enables unprecedented ability to access and manipulate the near-field coming from a light emitter or a scattering source^5,6^. HMMs can be composed of alternating metal and dielectric layers, of an array of metallic nanowires embedded in a dielectric and different other 2D and 3D metal-dielectrics^7^.

The aim of the present paper is to investigate the fundamental properties of surface plasmon modes localized at the planar interface between an arbitrary semi-infinite medium, metal or dielectric, with a semi-infinite metallodielectric superlattice. The term superlattice is used to emphasize the periodic arrangement of alternating metal and dielectric layers. Because of the analogy with electronic states localized at crystal lattice interfaces, we refer to these surface waves as Tamm plasmon modes (TPM). Surface optical waves at an interface between a metal and a purely dielectric superlattice (i.e. dielectric Bragg mirror), here referred to as Bragg TPMs, have been considered previously^8–10^. The fact that such Tamm plasmons appear within the dielectric light cones in both s- and p-polarization, inside the band gap of the Bragg mirror, makes them interesting for applications in lasers^11^, photodetectors^12^, engineering of spontaneous optical emission^10^ and chemical and biological refractometric sensors^13,14^.

In contrast to Bragg TPMs, the dispersion curves of metallodielectric TPMs have not been discussed systematically in literature so far. Metallodielectric TPMs are related to the traditional surface plasmon polaritons (SPPs) in that they are in-plane, p-polarized evanescent waves, but they stem from the hybridization of many single-interface SPPs of the metallodielectric superlattice and dielectric gap polaritons. The TPMs lie outside the dielectric light cone like the conventional SPPs and thus cannot escape from the flat interface, they appear when both the dielectric and metal layers are deeply subwavelength and have finite lateral group velocities. An important difference between Bragg and metallodielectric TPMs, perhaps crucial for tailoring spontaneous emission, is that the later exist even at the interface with air while the former are always buried beneath the interface with the Bragg mirror.

The current relevance of metallodielectric TPMs comes from recent reports on spontaneous emission enhancement by metallodielectric superlattices^15–17^ from which it is evident that they strongly affect the photonic density of states of an interface although a systematic understanding of their role is still lacking.

We analyze the conditions for the TPM existence for both metallic and dielectric capping layers, and determine their dispersion, propagation lengths as well as the TPM resonance strength quantified by the reflection coefficient residue at the TPM pole, which is proportional to the power a point dipole placed close to the interface would emit into the mode^18^. We also analyze the predictions made within the effective medium approximation (EMA), which becomes accurate in the limit of vanishing layer thicknesses.

### Two-phase stratified systems

We start with a reasoning requiring the use of single-interface boundary conditions only with minimal technical details. In spite of its deceptively simple appearance, it allows us to reach a general result regarding the existance of a particular surface mode in an arbitrary two-phase metallodielectric system and offers the explanation for some surprisingly simple properties of homointerface TPMs. A rigorous transfer-matrix-based method is then used in the remainder of the paper to confirm the general statements of this section for the particular case of a two-phase system involving a semi-infinite metallodielectric superlattice whose periodicity allows us to find closed-form solutions at the homointerface.

Starting from macroscopic Maxwell equations in which a dielectric and metallic medium are characterized by relative dielectric permittivities *ε*_d_ and *ε*_m_, the boundary conditions imposed on a p-polarized surface wave on the planar interface between two media, with transverse wavevector components *q*_*i*_ (*i* = d, m), are found to imply^2^

1 $${\alpha }_{{\rm{d}}}+{\alpha }_{{\rm{m}}}=\mathrm{0,}\quad {\alpha }_{i}=\frac{{q}_{i}}{{\varepsilon }_{i}},\quad i={\rm{d}},{\rm{m}}{\rm{.}}$$

**Figure 1:**
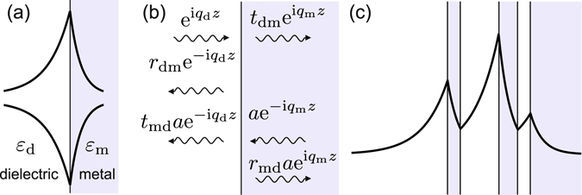
Schematics showing the general properties of stratified two-phase metallodielectric systems with an odd number of interfaces. (**a**) Two possible magnetic field variations which fulfill the interface boundary conditions. (**b**) Definition of Fresnel reflection and transmission coefficients. (**c**) Magnetic field of a mode fulfilling both the interface and boundary conditions at infinity.

Assuming that the *z*-axis is perpendicular to the interface and oriented towards the metallic medium, the fields of the surface wave are proportional to exp(−i*q*_d_*z*) and exp(i*q*_m_*z*), in the dielectric and metal medium respectively, from which it follows that the imaginary parts of both *q*_*i*_ must be positive in order to meet boundary conditions at infinity. This case corresponds to a magnetic field whose magnitude has a peak at the interface, as indicated by the top curve in Fig. 1(a).

However, Eq. (1) remains fulfilled if *q*_*i*_ are both replaced by −*q*_*i*_, corresponding to fields growing exponentially away from the local minimum at interface, as depicted by the bottom curve in Fig. 1(a).

Introducing the Fresnel reflection and transmission coefficients as

2 $${r}_{{\rm{dm}}}=\frac{{\alpha }_{{\rm{d}}}-{\alpha }_{{\rm{m}}}}{{\alpha }_{{\rm{d}}}+{\alpha }_{{\rm{m}}}},\quad {t}_{{\rm{dm}}}=1+{r}_{{\rm{dm}}},$$

for incidence from the dielectric side, and

3 $${r}_{{\rm{md}}}=-{r}_{{\rm{dm}}},\quad {t}_{{\rm{md}}}=1+{r}_{{\rm{md}}}=1-{r}_{{\rm{dm}}},$$

for the opposite incidence, the case of field having the peak at the interface is seen to represent the scattered field for an incoming wave from either medium, when |*r*_dm_| diverges, which is equivalent^19^ to Eq. (1).

The analogous explanation of how the solution growing away from the interface arises, is found by assuming two waves are incident on the interface, the one from the dielectric side with unit amplitude and the other with complex amplitude *a*, as depicted in Fig. 1(b). By chosing *a* = *r*_dm_/(*r*_dm_ − 1), the fields going away from the interface cancel out when *r*_dm_ has a pole, leaving only the incoming components which correspond to the exponential growth away from the interface.

The significance of the latter solution to interface boundary conditions becomes apparent when additional layers with permittivities *ε*_d_ and *ε*_m_ are inserted between the semi-infinite dielectric and metal media, which means adding an even number of interfaces to the initial one. Now it is seen that if the boundary conditions at each interface are satisfied by alternating field maxima and minima, as depicted in Fig. 1(c), the boundary conditions at infinity will also be fulfilled, meaning that the obtained field will represent a surface mode of the multilayer. Therefore, any metallodielectric multilayer capped by the semi-infinite dielectric on one side and the metallic medium on the other, will have at least one surface mode whose transverse wavevector components fulfill Eq. (1), which implies that its dispersion curve is identical with the single-interface SPP dispersion curve

4 $${\beta }_{{\rm{dm}}}={k}_{0}\sqrt{\frac{{\varepsilon }_{{\rm{d}}}{\varepsilon }_{{\rm{m}}}}{{\varepsilon }_{{\rm{d}}}+{\varepsilon }_{{\rm{m}}}}},$$

where *β*_dm_ represents the complex amplitude of the longitudinal (parallel to interfaces) wavevector component and *k*_0_ = *ω*/*c* is the free-space wavenumber.

**Figure 2:**
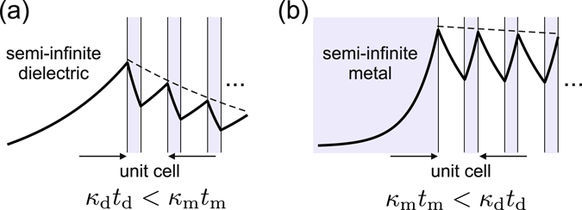
Magnetic field variation on homointerfaces for (**a**) dielectric and (**b**) metallic capping medium. The requirement that the Bloch anvelope, indicated by dashed lines, decays towards infinity on the right, yield the condition that *κ*_d_*t*_d_ < *κ*_m_*t*_m_ and *κ*_d_*t*_d_ > *κ*_m_*t*_m_ for (**a**,**b**), respectively. Note that the values of *κ*_d_ and *κ*_m_ used in panels (**a**,**b**) differ.

To see the implications of this general result for semi-infinite metallodielectric superlattices, we consider a surface wave, henceforth referred to as TPM, with a dispersion given by Eq. (4). The first condition it has to satisfy on a semi-infinite superlattice capped by a dielectric, as depicted in Fig. 2(a), is that it decays exponentially to the left, meaning that moving to the right, it has to decay in metallic layers and grow in dielectric ones. Denoting by *t*_*i*_ the layer thicknesses and *κ*_*i*_ the imaginary part of *q*_*i*_, we see that the field amplitude is multiplied by exp(−*κ*_m_*t*_m_) and exp(*κ*_d_*t*_d_) in passing through the metal and the dielectric layer, respectively, meaning that passing through one unit cell, its amplitude is multiplied by exp(−*κ*_m_*t*_m_ + *κ*_d_*t*_d_). The second condition imposed on this TPM is that it decays towards infinity on the right side, therefore a TPM on the dielectric-capped superlattice can exist only if

5 $${\kappa }_{{\rm{d}}}{t}_{{\rm{d}}} < {\kappa }_{{\rm{m}}}{t}_{{\rm{m}}}\mathrm{.}$$

Repeating the above reasoning for the metal-capped superlattice, the field amplitude is seen to be multiplied by exp(*κ*_m_*t*_m_ − *κ*_d_*t*_d_) in passing each unit cell, so a TPM with dispersion given by Eq. (4) will exist if the opposite is true

6 $${\kappa }_{{\rm{d}}}{t}_{{\rm{d}}} > {\kappa }_{{\rm{m}}}{t}_{{\rm{m}}}\mathrm{.}$$

In the following section we show rigorously that the homointerface supports only the TPMs given by Eq. (4), in conjunction with conditions (5) or (6), depending on the capping medium. Under such circumstances, the reasoning applied in this section has a purpose of giving an intuitive explanation of several coincidences that might not have been expected. One interesting point to note here is that if the two possible homointerfaces of a given semi-infinite superlattice are considered, one being capped by the dielectric and the other by the metal constituent medium as in Fig. 2(a) and (b), at any given frequency *ω* only one of the conditions in Eqs. (5) or (6) can be fulfilled, so only one of them can support a TPM. This hints that in drawing the schematics in Fig. 2, different values of *ω* have been assumed for panels (a) and (b).

### Homointerface

**Figure 3:**
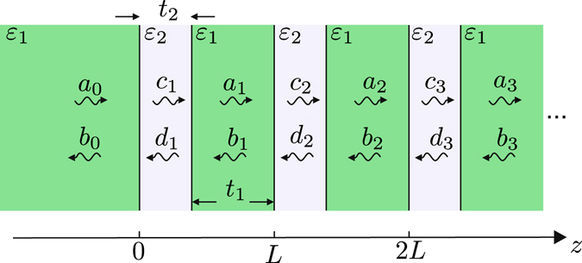
Schematics of the homointerface geometry used in the transfer-matrix method. The *ε*_1_ medium extends from left infinity up to the interface at *z* = 0, after which the superlattice is formed by periodically repeating the unit cell comprising a *ε*_2_ and *ε*_1_ layer.

The general geometry of the problem is sketched in Fig. 3. The capping medium permittivity is assumed equal to one of the lattice constituents, *ε*_1_, while the second medium permittivity is *ε*_2_. This system will henceforth be referred to as homointerface. In determining the fields, we follow the transfer-matrix method of ref.^20^ For completeness and due to some minor differences in notation, here we briefly summarize the main aspects of the method.

The trasverse field phasor *F* in the *n*-th unit cell is represented as a sum of the forward and backward propagating plane wave amplitudes, denoted by *a*_*n*_ and *b*_*n*_ in medium *ε*_1_ and by *c*_*n*_ and *d*_*n*_ in the second medium. *F* corresponds to the electric or magnetic field for a s- or p-polarized wave, respectively. The wave amplitudes *a*_*n*_, *b*_*n*_ of adjacent unit cells are related by the translation matrix

7 $$[\begin{array}{c}{a}_{n-1}\\ {b}_{n-1}\end{array}]=T[\begin{array}{c}{a}_{n}\\ {b}_{n}\end{array}],\quad T=[\begin{array}{cc}A & B\\ C & D\end{array}],$$

whose elements are given by

8 $$A={{\rm{e}}}^{-{\rm{i}}{q}_{1}{t}_{1}}[\cos ({q}_{2}{t}_{2})-\frac{{\rm{i}}}{2}(\frac{{\alpha }_{2}}{{\alpha }_{1}}+\frac{{\alpha }_{1}}{{\alpha }_{2}})\sin ({q}_{2}{t}_{2})],$$

9 $$B=-{{\rm{e}}}^{{\rm{i}}{q}_{1}{t}_{2}}\frac{{\rm{i}}}{2}(\frac{{\alpha }_{2}}{{\alpha }_{1}}-\frac{{\alpha }_{1}}{{\alpha }_{2}})\sin ({q}_{2}{t}_{2}),$$

10 $$C={{\rm{e}}}^{-{\rm{i}}{q}_{1}{t}_{2}}\frac{{\rm{i}}}{2}(\frac{{\alpha }_{2}}{{\alpha }_{1}}-\frac{{\alpha }_{1}}{{\alpha }_{2}})\sin ({q}_{2}{t}_{2}),$$

11 $$D={{\rm{e}}}^{{\rm{i}}{q}_{1}{t}_{1}}[\cos ({q}_{2}{t}_{2})+\frac{{\rm{i}}}{2}(\frac{{\alpha }_{2}}{{\alpha }_{1}}+\frac{{\alpha }_{1}}{{\alpha }_{2}})\sin ({q}_{2}{t}_{2})]\mathrm{.}$$

Here *q*_*i*_ represent the complex wavector *z*-components, related with the longitudinal (in-plane) component *β* via the dispersion relation of each layer

12 $${q}_{i}=\pm \sqrt{{\varepsilon }_{i}{k}_{0}^{2}-{\beta }^{2}},\quad {\rm{Im}}\{{q}_{i}\}\ge \mathrm{0,}$$

with ± denoting the sign that gives *q*_*i*_ with a non-negative imaginary part. In case of s-polarization, *α*_*i*_ = *q*_*i*_ while for p-polarization *α*_*i*_ = *q*_*i*_/*ε*_*i*_, as before.

From the above, *T* is seen to be unimodular for any (real or complex) *ε*_*i*_ or *q*_*i*_. It further implies that the product of its two eigenvalues is unity, so they can be written in the form e^±i*KL*^, where *L* = *t*_1_ + *t*_2_ and *K* is the Bloch wavenumber. Here we define *K* so that exp(i*KL*) corresponds to a wave propagating towards positive infinity, meaning that

13 $${{\rm{e}}}^{{\rm{i}}KL}=\frac{A+D}{2}\pm \sqrt{{(\frac{A+D}{2})}^{2}-\mathrm{1,}}\quad {\rm{Im}}\{K\}\ge \mathrm{0,}$$

where ± denotes the sign giving a non-negative imaginary part of *K*.

Before proceeding further, the basic aspects of dissipation should be clarified. At visible and infrared frequencies, the dominant source of dissipation, by far, are intra- and inter-band electronic transitions in the metal. Therefore, we assume that the dielectric has a purely real relative permittivity *ε*_d_ = 6.76, corresponding to titanium dioxide, while for metal we use the silver Drude-Lorentz parametrized experimental data from ref.^21^. A convenient method^22^ to treat the problem of losses is to introduce a modified dielectric function of the metal via the perturbation parameter *p*

14 $${\varepsilon }_{{\rm{m}}}^{(p)}={\rm{Re}}\{{\varepsilon }_{m}\}+{\rm{i}}p{\rm{Im}}\{{\varepsilon }_{m}\},\quad {\varepsilon }_{{\rm{m}}}^{\mathrm{(1)}}\triangleq {\varepsilon }_{{\rm{m}}}\mathrm{.}$$

Setting *p* to unity recovers the original *ε*_m_, while reducing it towards zero approaches smoothly the lossless case ${\varepsilon }_{{\rm{m}}}^{\mathrm{(0)}}$ in which the optical eigenmodes can properly be defined. The latter are thus used as a convenient scaffold on which quantities calculated with real loss are projected. The eigenmodes of a lossless infinite superlattice, henceforth referred to as bulk modes, are defined as solutions of Eq. (13) having both their eigenfrequency *ω* and wavevector **k** = (**β**, *K*) purely real.

**Figure 4:**
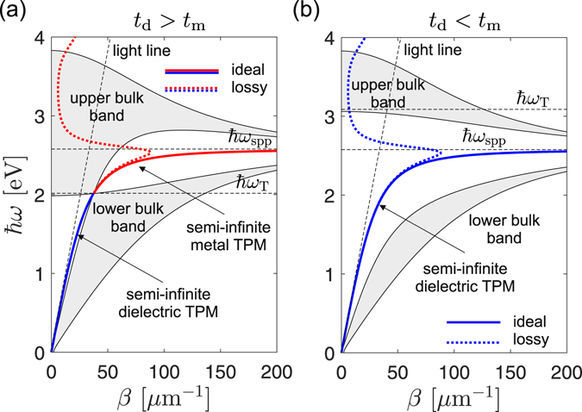
Bulk band structure (shading) and TPMs (thick lines) of one typical example for each the two possible band structure topologies. (**a**) *t*_d_ > *t*_m_ with *ω*_T_ < *ω*_spp_, leading to an intersection of the bands at *ω*_T_. Here titanium dioxide and silver are assumed with *t*_d_ = 20 nm and *t*_m_ = 10 nm. (**b**) *t*_d_ > *t*_m_ leading to *ω*_T_ < *ω*_spp_ meaning that the upper and lower bulk bands do not intersect. Here *t*_d_ = 10 nm and *t*_m_ = 20 nm is assumed for titanium dioxide and silver, respectively.

Depending on the relative thickness of the dielectric, *t*_d_, and metal, *t*_m_, layers, the band structure of the infinite superlattice is known to have one of two possible topologies, corresponding to *t*_d_ > *t*_m_ and *t*_d_ < *t*_m_, as indicated by the shaded areas in Fig. 4(a) and (b). In this paper we carry out concrete calculations for one representative example from both classes: *t*_d_ = 20 nm, *t*_d_ = 10 nm, shown in Fig. 4(a), and *t*_d_ = 10 nm, *t*_m_ = 20 nm, in Fig. 4(b).

A general property of the *t*_d_ > *t*_m_ class is that the upper and lower bulk bands intersect^23^ at a frequency *ω*_T_ we shall refer to as the transition frequency. In the literature, *ω*_T_ is sometimes^15^ referred to as the topological transition point since the band topology changes when crossing *ω*_T_. It can be shown^23^ that *ω*_T_ is defined by the condition

15 $${t}_{{\rm{d}}}{\varepsilon }_{{\rm{d}}}+{t}_{{\rm{m}}}{\varepsilon }_{{\rm{m}}}({\omega }_{{\rm{T}}})=\mathrm{0,}$$

which can alternatively be stated as

16 $${\varepsilon }_{\parallel }({\omega }_{{\rm{T}}})=\mathrm{0,}$$

with *ε*_||_ denoting the average in-plane permittivity (or the in-plane permittivity tensor component in EMA)

17 $${\varepsilon }_{\parallel }=\eta {\varepsilon }_{1}+\mathrm{(1}-\eta ){\varepsilon }_{2},\quad \eta =\frac{{t}_{1}}{L}\mathrm{.}$$

While it is obvious that *ω*_T_ can be engineered by varying the thickness of layers in the metallodielectric superlattice, it is interesting that its value does not depend on the actual layer thicknesses but only on their ratio *t*_d_/*t*_m_.

The bulk bands of the *t*_d_ < *t*_m_ class are separated by *β*_dm_(*ω*) which lies between them. The band edges shown by thin (black) lines in Fig. 4 correspond to solutions having cos(*KL*) = 1 (inner boundaries) and cos(*KL*) = −1 (outer boundaries). In a lossy system, however, there is no strict definition of band edges, but rather a continuum of modes in the *β* − *ω* plane whose lifetime and propagation lengths increase rapidly around the band edges of the lossless system^22^.

Having defined the bulk bands of the superlattice, we are now ready to discuss the surface modes of the semi-infinite superlattice, i.e. the TPMs. By definition, the surface mode at a planar interface must be located outside the bulk bands of both the capping medium and the superlattice, meaning that it must be represented by a point in the *β* − *ω* plane outside both the light line and the shaded regions in Fig. 4.

TPMs are identified as poles of the reflection coefficient *r* of an incoming wave from the capping layer. The reflection coefficient *r* for the semi-infinite superlattice is determined as the *N* → ∞ limit of the reflection coefficient *r*_*N*_ of a system comprising *N* unit cells, such that the *ε*_1_ medium of the *N*-th unit cell is semi-infinite. In that case^20^, *a*_*N*_, *b*_*N*_ are related with *a*_0_, *b*_0_ via *T*^*N*^ while *r*_*N*_ is defined as the ratio of *b*_0_ and *a*_0_ when there is no incoming wave in the *N*-th layer. Using the known expression^24^ for *T*^*N*^, we find

18 $${r}_{N}=\frac{C}{A-\,\sin ([N-\mathrm{1]}KL)/\,\sin (NKL)},$$

with *A* and *C* given by Eqs. (8) and (10). Remembering that *K* has at least an infinitesimaly small positive imaginary part, we find

19 $$\mathop{\mathrm{lim}}\limits_{N\to \infty }\frac{\sin ([N-\mathrm{1]}KL)}{\sin (NKL)}={{\rm{e}}}^{{\rm{i}}KL},$$

and finally

20 $$r=\mathop{\mathrm{lim}}\limits_{N\to \infty }{r}_{N}=\frac{C}{A-{{\rm{e}}}^{{\rm{i}}KL}}\mathrm{.}$$

The condition for the existence of a TPM is that *r* diverges which happens when

21 $$A={{\rm{e}}}^{{\rm{i}}KL}\mathrm{.}$$

Combining Eq. (13) with Eqs. (8) and (11), the necessary condition for Eq. (21) to hold in a non-trivial case is found to be

22 $${\alpha }_{1}+{\alpha }_{2}=0.$$

Since this is equivalent with Eq. (1), a TPM can exist only in p-polarization while its in-plane wavenumber must be qual to that of the single-interface SPP *β*_TPM_(*ω*) = *β*_md_(*ω*).

Combining Eqs. (21) and (22) for p-polarization gives

23 $$K=\frac{{q}_{2}{t}_{2}-{q}_{1}{t}_{1}}{L}=-{\alpha }_{1}{\varepsilon }_{\parallel }={\alpha }_{2}{\varepsilon }_{\parallel }\mathrm{.}$$

Since *K* has a non-negative imaginary part by definition, we find that a TPM can exist on a homointerface only if

24 $${\rm{Im}}\{-{\alpha }_{1}{\varepsilon }_{\parallel }\}={\rm{Im}}\{{\alpha }_{2}{\varepsilon }_{\parallel }\}\ge 0.$$

For a dielectric-capped homointerface, where *ε*_1_ = *ε*_d_, Eq. (24) requires *ε*_|__|_ to be negative in the lossless limit, meaning that the TPM exist only for frequencies up to *ω*_T_, as indicated by the thick solid blue curves in Fig. 4(a) and (b). In contrast, the metal-capped homointerface, where *ε*_1_ = *ε*_m_, supports a TPM only above *ω*_T_ as *ε*_||_ must be positive. As *ω*_T_ is below the surface plasmon resonance frequency *ω*_spp_ (the upper frequency limit for SPP propagation), only if *t*_d_ > *t*_m_, a TPM on a metal-capped homointerface exists only in the *t*_d_ > *t*_m_ class of superlattices, as indicated by the thick solid red line in Fig. 4(a).

Solving for Eqs. (22) and (24) with loss taken into account yields complex values for *β*_TPM_(*ω*), the real part of which is drawn in Fig. 4(a) and (b) by thick dotted lines. We find that, quite generally, at any given frequency *ω* a TPM mode with *β*_TPM_(*ω*) = *β*_md_(*ω*) exists either on the metal- or dielectric capped superlattice, which is manifested by the fact that taken together, they form the complete SPP dispersion curves *β*_dm_(*ω*) in Fig. 4(a) and (b). In the lossy case, some subtleties arise regarding the inequality in Eq. (24), as indicated by the fact that the metal-capped *t*_d_ > *t*_m_ homointerface is found to support a TPM above around 3.82 eV. Such modes, however, exist only formally as their lifetimes are way too short to be relevant.

The above transfer-matrix-based homointerface TPM analysis thus shows formally that the TPM eigenmodes discussed on general grounds in Section "Two-phase stratified systems" are, in fact, the only possible surface modes and that they have properties very similar to single-interface SPPs. Before proceeding with details showing the important differences between the two, we briefly look at the problem from the EMA perspective.

In EMA, the layer thicknesses *t*_*i*_ are assumed to be negligible relative to any length scale relevant for wave propagation, so the superlattice is described by two dielectric permittivities *ε*_||_ and *ε*_⊥_, with the former having been defined previously in Eq. (17) and the latter given by

25 $${\varepsilon }_{\perp }=\frac{{\varepsilon }_{1}{\varepsilon }_{2}}{\mathrm{(1}-\eta ){\varepsilon }_{1}+\eta {\varepsilon }_{2}}\mathrm{.}$$

The reflection coefficient for a p-polarized wave incident from a semi-infinite medium *ε*_1_ is

26 $${r}_{{\rm{ema}}}=\frac{{\alpha }_{1}-{\alpha }_{{\rm{ema}}}}{{\alpha }_{1}+{\alpha }_{{\rm{ema}}}},\quad {\alpha }_{{\rm{ema}}}=\frac{{q}_{{\rm{ema}}}}{{\varepsilon }_{\parallel }},$$

while the transverse wavenumber in the effective uniaxial medium is

27 $${q}_{{\rm{ema}}}=\pm \sqrt{{\varepsilon }_{\parallel }{k}_{0}^{2}-\frac{{\varepsilon }_{\parallel }}{{\varepsilon }_{\perp }}{\beta }^{2}},\quad {\rm{Im}}\{{q}_{{\rm{ema}}}\}\ge 0.$$

The dispersion of the surface mode at the interface between *ε*_1_ and the uniaxial medium is obtained as

28 $${\beta }_{{\rm{ema}}}=\pm {k}_{0}\sqrt{\frac{{\varepsilon }_{1}{\varepsilon }_{\perp }({\varepsilon }_{\parallel }-{\varepsilon }_{1})}{{\varepsilon }_{\parallel }{\varepsilon }_{\perp }-{\varepsilon }_{1}^{2}}},\quad {\rm{Im}}\{{\beta }_{{\rm{ema}}}\}\ge 0.$$

Replacing *ε*_||_ and *ε*_⊥_ by expressions given by Eqs. (17) and (25), we find that *β*_ema_ = *β*_dm_. Since the divergence of *r*_ema_ requires *q*_ema_ = −*α*_1_*ε*_||_, a comparison with Eq. (23), shows that the perpendicular component of the wave in EMA is equal to the Bloch wavenumber

29 $$K={q}_{{\rm{ema}}}\mathrm{.}$$

Therefore, EMA describes correctly not only the dispersion and condition for the existence of the TPM, but also its transverse extension into the superlattice quantified by the penetration depth

30 $$\delta =\frac{1}{{\rm{Im}}\{K\}}=\frac{1}{{\rm{Im}}\{{q}_{{\rm{ema}}}\}}\mathrm{.}$$

The overlap of complex dispersion curves *β*_dm_(*ω*), *β*_TPM_(*ω*) and *β*_ema_(*ω*), corresponding to the single-interface SPP, TPM and the EMA surface wave, is somewhat unexpected considering that it implies an idential modal decay dynamics which is known to be determined by how is the modal energy distributed in space^22^. The fact that these three modes have a very different field variation in the direction perpendicular to the interface while sharing the same dispersion curve is, evidently, a consequence of the simple rule by which the boundary conditions in a two-phase stratified system can be fulfilled, as discussed in Section "Two-phase stratified systems", which allows the ratio of the total field energy residing in the dielectric and metal medium to remain invariant upon the insertion of an arbitrary number interfaces (as long as their total number is odd).

The manner in which the presence of a surface mode modifies the optical properties of an interface is determined by how the reflection coefficient *r*(*β*), considered as a function over the complex *β* plane, behaves in the vicinity of the associated pole *β*_pole_. For example, ref.^18^ shows that the power emitted by a dipole located in the vicinity of a metal-dielectric interface is proportional to the residue *a*_pole_ of *r*(*β*) at *β*_pole_, evaluated as

31 $${a}_{{\rm{pole}}}=\frac{1}{2\pi {\rm{i}}}{\oint }_{\gamma }\frac{r(\beta )d\beta }{\beta -{\beta }_{{\rm{pole}}}},$$

where *γ* denotes a positively oriented contour around *β*_pole_ in the complex *β*-plane, sufficiently small so that *r*(*β*) is analytic within it (i.e. avoiding the branch cuts associated with *q*_*i*_).

The analytic expression for the single-interface SPP residue has been reported in ref.^18^

32 $${a}_{{\rm{spp}}}=\frac{2{\varepsilon }_{1}{\varepsilon }_{2}}{{\varepsilon }_{1}^{2}-{\varepsilon }_{2}^{2}}{\beta }_{{\rm{sp}}}\mathrm{.}$$

Since the homointerface TPM and the EMA surface wave dispersion curves overlap with the SPP, it is straightforward to show that their residues are given by

33 $${a}_{{\rm{TPM}}}=\frac{\sin ({q}_{2}{t}_{2}-{q}_{1}{t}_{1})}{\exp ({\rm{i}}{q}_{1}{t}_{1})\sin ({q}_{2}{t}_{2})}{a}_{{\rm{spp}}},$$

where *q*_1_ and *q*_2_ should be evaluated at *β*_TPM_, and

34 $${a}_{{\rm{ema}}}=(1+\frac{f}{1-f}\frac{{\varepsilon }_{1}}{{\varepsilon }_{2}}){a}_{{\rm{spp}}},$$

respectively. The last two equations hold only if the mode exists while in case it does not, the corresponding residue is zero.

**Figure 5:**
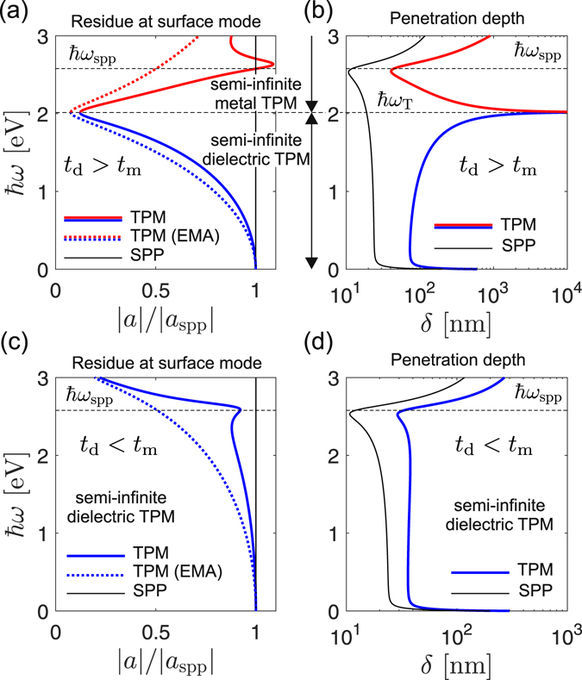
Left panels: residue at the TPM and SPP mode, drawn for the (**a**) *t*_d_ > *t*_m_ and (**c**) *t*_d_ < *t*_m_ examples, respectively. Righ panels: penetration depths into the superlattice for (**a**) *t*_d_ > *t*_m_ and (**c**) *t*_d_ < *t*_m_ examples, compared with the corresponding SPP penetration depth.

Comparing the magnitudes of *a*_spp_, *a*_TPM_ and *a*_ema_ in a *t*_d_ > *t*_m_ type dielectric-capped superlattice, both |*a*_TPM_| and |*a*_ema_| are seen to decrease monotonously as *ω* is increased towards *ω*_T_, Fig. 5(a) (blue line). This happens because the argument of the sine function in *a*_TPM_ in Eq. (33) is, according to Eq. (23), proportional to *ε*_||_ and thus decreases close to zero (or becomes exactly zero in the lossless limit) at *ω*_T_. Since |*a*_ema_| is the limit of |*a*_TPM_| for vanishing layer thicknesses (this holds by definition of EMA and is also evident by taking the *t*_*i*_ → 0 limit of Eq. (33)) it is depicted by dotted lines and indicates the possible trend of the |*a*_TPM_| spectra in case the layer thicknesses are scaled down. Above *ω*_*T*_, the TPM mode of the dielectric-capped homointerface disappears, meaning that |*a*_TPM_| drops to zero. In order to simplify Fig. 5, the curves corresponding to |*a*_TPM_| = 0 are omitted, so in Fig. 5(a) the blue and red curves are discontinued at *ω* = *ω*_T_. In the lossless limit (not shown), |*a*_TPM_| becomes exactly zero at *ω*_T_.

The opposite trend of |*a*_TPM_| is observed for the *t*_d_ > *t*_m_ type metal-capped superlattice, where |*a*_TPM_| starts from small values (zero in the lossless limit) at *ω*_T_ and approaches |*a*_spp_| as *ω* goes to *ω*_spp_. The disappearance of the TPM resonance around *ω*_T_ is accompanied with a delocalization of the mode energy across the superlattice, as seen in Fig. 5(b). In the lossless limit, the penetration depth *δ* of the TPM mode diverges at *ω*_T_, while in the real lossy case shown in Fig. 5(b), *δ* reaches values up to around 10 microns. The penetration depth of the single-interface SPP shown for reference (black line), varies only a bit between 20 and 30 nm over the infrared and visible frequencies, up to *ω*_spp_ where it has a dip.

For the *t*_d_ < *t*_m_ class of superlattices, a simpler behavior is found, as depicted in Fig. 5(c) and (d). Here |*a*_TPM_| is only slightly below |*a*_spp_|, while the TPM penetrates only slightly more into the superlattice, showing no pronounced spectral variations.

Figures 5(a) and (c) show that the EMA description of homointerface TPMs is not entirely accurate, after all. The fact that *a*_ema_ differs from *a*_TPM_ means that EMA does not account properly for the TPM contribution to the reflection coefficient, meaning the it will give an erroneous TPM dispersion if any additional interface is added, as it is the case with a heterointerface.

### Heterointerface

**Figure 6:**
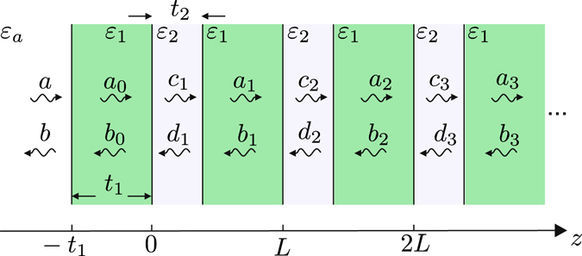
Schematics of the heterointerface geometry. It is obtained from the homointerface geometry in Fig. 4 by replacing the semi-infinite *ε*_1_ medium with a semi-infinite medium *ε*_*a*_ extending from left infinity up to *z*  = −*t*_1_, so that the structure remains invariant for *z* > −*t*_1_. In the text we show that the TPM properties are determined by a combined interaction of the newly added *ε*_*a*_ − *ε*_1_ interface at *z* = −*t*_1_ and the homointerface at *z* = 0 buried under the top *ε*_1_ layer of the superlattice.

A heterointerface is obtained when the capping medium permittivity *ε*_*a*_ differs from the permittivities *ε*_1_, *ε*_2_ of the two superlattice constituents, as shown in Fig. 6. The notation is chosen so that *ε*_1_ denotes the permittivity of the top layer of the superlattice, which is shown below to play a major role in determining the types of TPMs supported by the interface. Denoting by *a* and *b* the plane wave amplitudes of the incoming and scattered wave, the heterointerface reflection coefficient *r*_het_ is defined as their ratio for excitation from the capping medium. Introducing the auxiliary reflection coefficient

35 $${r}_{a}=\frac{{\alpha }_{a}-{\alpha }_{1}}{{\alpha }_{a}+{\alpha }_{1}},$$

which corresponds to reflection on a single interface between *ε*_*a*_ and *ε*_1_, the total reflection coefficient is obtained as

36 $${r}_{{\rm{het}}}=\frac{{r}_{a}+r\exp \mathrm{(2}{\rm{i}}{q}_{1}{t}_{1})}{1+{r}_{a}r\exp \mathrm{(2}{\rm{i}}{q}_{1}{t}_{1})}\mathrm{.}$$

The condition for the existence of a TPM mode in the non-trivial case (*r*_*a*_ ≠ 0 and *r* ≠ 0) is that the denominator vanishes

37 $$1+{r}_{a}r\exp \mathrm{(2}{\rm{i}}{q}_{1}{t}_{1})=\mathrm{0,}$$

where we recognize the typical condition for a cavity resonance, with *r*_*a*_ and *r* being the mirror reflection coefficients. In ultrathin films considered here, the real part of phase 2*q*_1_*t*_1_ is much below *π*, meaning that the heterointerface TPMs are not expected to have a Fabri-Perot character but to be formed by the modification of poles of either *r*_*a*_ or *r*, as the rapid variation of the reflection phase in their vicinity allows the condition in Eq. (37) to be satisfied.

For a given frequency *ω*, the complex in-plane wavenumber of a TPM fulfilling Eq. (37) will be denoted by *β*_*n*_(*ω*), with *n* = 1, 2, … enumerating the existing TPMs. In contrast to the homointerface case in which only one TPM is allowed while the dependence of *β*_TPM_ on *ω* is given by an analytic expression, here multiple solutions may exist while *β*_*n*_(*ω*) cannot, in principle, be expressed analytically. Therefore, we look for TPM modes by evaluating *r*_het_(*ω*, *β*) in the *β* − *ω* plane^19^ and use the fact that the local density of optical states *ρ*(*ω*, *β*) at the interface is proportional^25^ to the imaginary part of *r*_het_, so that sharp maxima of |Im{*r*_het_}| signal the presence of a TPM.

**Figure 7:**
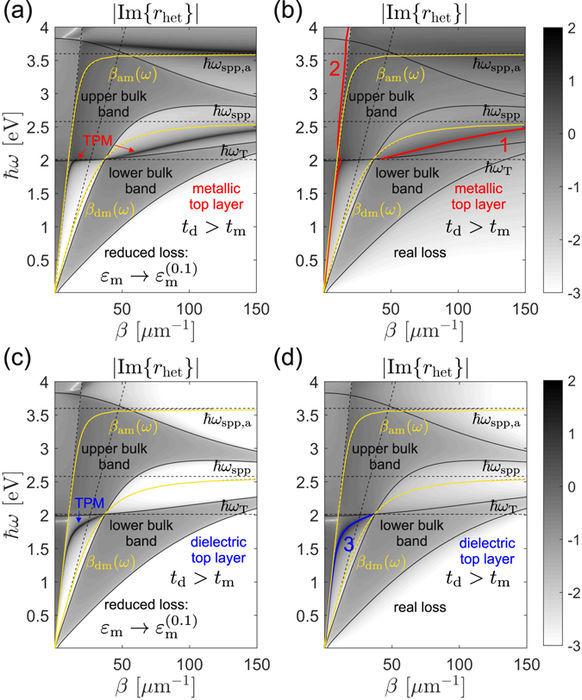
Maps of |Im{*r*_het_}| drawn in logarithmic scale with the colormap chosen so that all values outside the (−3, 2) interval are saturated. Top panels are drawn for superlattices terminated by a metallic layer (*ε*_1_ = *ε*_m_), while the bottom ones correspond to a dielectric top layer (*ε*_1_ = *ε*_d_). Left panels: maps obtained by multiplying the imaginary part of the silver permittivity by *p* = 0.1 in order to sharpen the features and render TPMs visible. Right panels: maps obtained by taking the loss fully into account. All maps correspond to the *t*_d_ > *t*_m_ example. The two slanted dashed lines represent the *ε*_*a*_ and *ε*_d_ light lines.

Typical *r*_het_ maps for *t*_d_ > *t*_m_ heterointerface types are depicted in Fig. 7(a–d), with lossless single-interface ${\varepsilon }_{{\rm{d}}}-{\varepsilon }_{{\rm{m}}}^{\mathrm{(0)}}$ and ${\varepsilon }_{a}-{\varepsilon }_{{\rm{m}}}^{\mathrm{(0)}}$ SPP dispersion curves denoted by *β*_md_(*ω*) and *β*_am_(*ω*) drawn for reference. In order to sharpen the map features, we compare the maps corresponding the actual silver permittivity *ε*_m_ (right panels of Fig. 7) with ones obtained by assuming reduced losses, i.e. ${\varepsilon }_{{\rm{m}}}^{(p)}$ with *p* < 1. Here we use *p* = 0.1, as it is found to be sufficient for reliably resolving the TPM dispersion in the *β* − *ω* plane. As expected, the |Im{*r*_het_}| has higher values within the bands, and decreases rapidly with crossing the band edges indicated by thin (black) solid lines. The TPM modes show up as dark bands in the *β* − *ω* plane outside of the bulk bands.

A formal justification for relating the properties of the two maps is based on perturbation theory^22^ which shows that, up to the first order, the real part of the modal wavenumber Re{*β*_*n*_} is independent on the perturbation parameter *g* = Im{*ε*_m_}/*ε*_m_, while Im{*β*_*n*_} has a linear proportionality. Therefore, the sharp maps in the left panels of Fig. 7 are used for estimating the complex *β*_*n*_. The estimated value is then used as a starting guess for a Nelder-Mead minimization method^26^ implemented numerically, which finds the solutions for *β*_*n*_, with high accuracy, as the minima of the absolute value of the left-hand side in Eq. (37).

In the case of a metallic top layer of the *t*_d_ > *t*_m_ heterointerface, two TPM modes, TPM-1 and TPM-2, are identified, as indicated by thick enumerated (red) lines in Fig. 7(b). The curves representing Re{*β*_1_(*ω*)} and Re{*β*_2_(*ω*)} are seen to correspond well to the sharp peaks in the vicinity of *β*_am_(*ω*) and *β*_md_(*ω*) curves in Fig. 7(a). At first, it might apear unusual that we do not consider the dark band in Fig. 7(a) appearing slightly above *ω*_spp,a_ and having a negative slope. We have found that, although formally a TPM mode, it is so far from the real *β*-axis that the associated propagation length is in the nanometer range, which makes it effectively indistinguishable from the air-silver surface plasmon resonance. The fact that this band appears as sharp in Fig. 7(a) as TPM-1 and TPM-2, while numerical calculations show it is dissipated much more, indicates that the perturbation picture does not work well above the top bulk band. Indeed, in the vicinity of *ω*_spp,a_ the real and the imaginary part of *ε*_m_ have comparable magnitudes, meaning that |*g*| is not small compared to unity as required by perturbation theory^22^.

The fact that TPM-1 and TPM-2 in Fig. 7(b) arise by a modification of the metal-capped homointerface TPM and the single-interface *ε*_*a*_ − *ε*_m_ SPP, respectively, can be shown by considering Eq. (37). Assuming first that |*r*_*a*_exp(2i*q*_1_*t*_1_)| is small around the *β*_TPM_(*ω*) pole of *r*, so that the *β*_1_(*ω*) pole of *r*_het_ is close to *β*_TPM_(*ω*), *r* can be written as

38 $$r(\omega ,\beta )\approx \frac{{a}_{{\rm{TPM}}}}{\beta -{\beta }_{{\rm{TPM}}}(\omega )},$$

which combined with Eq. (37) yields

39 $${\beta }_{1}(\omega )\approx {\beta }_{{\rm{TPM}}}(\omega )-{a}_{{\rm{TPM}}}{r}_{a}\exp \mathrm{(2}{\rm{i}}{q}_{1}{t}_{1}\mathrm{).}$$

This shows that if the heterointerface is obtained by gradually changing the capping permittivity from *ε*_1_ to *ε*_*a*_, the dispersion curve *β*_1_(*ω*) gradually evolves from *β*_TPM_(*ω*), with a deviation increasing as *r*_*a*_ increases and with a factor proportional to the homointerface TPM residue *a*_TPM_. Another important implication of Eq. (39) is that using EMA does not yield a correct dispersion of heterointerface TPMs because *a*_ema_ differs from *a*_TPM_.

Repeating a similar analysis but now assuming *β*_2_(*ω*) is in the vicinity of *β*_am_(*ω*), we find

40 $${\beta }_{2}(\omega )\approx {\beta }_{{\rm{am}}}(\omega )-{a}_{\mathrm{spp},a}r\exp \mathrm{(2}{\rm{i}}{q}_{1}{t}_{1}),$$

which shows that *β*_2_(*ω*) will be very close to *β*_am_(*ω*) if |*r* exp(2i*q*_1_*t*_1_)| is small which e.g. is a good approximation for *t*_1_ = *t*_m_ = 20 nm, or higher. Equations (39) and (40) thus allow us to classify the heterointerface TPMs into homointerface-like (TPM-1) and SPP-like (TPM-2).

The bottom two panels of Fig. 7 show the case in which the *t*_d_ > *t*_m_ superlattice is terminated by the dielectric layer. Here only one solution, TPM-3, exists which evidently originates from the dielectric-capped homointerface TPM meaning it falls under the homointerface-like TPM type.

The thick TPM dispersion curves in Fig. 7 show the real part of the modal in-plane wavenumber *β*_*n*_. The corresponding imaginary parts determine the lateral propagation length of the mode

41 $${L}_{\parallel ,n}=\frac{1}{{\rm{Im}}\{{\beta }_{n}\}}\mathrm{.}$$

**Figure 8:**
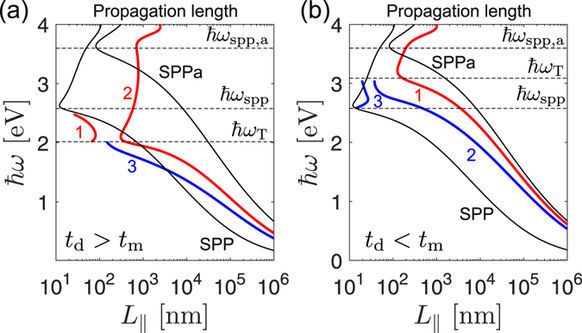
In-plane propagation length *L*_||_ for TPMs whose dispersion of Re{*β*_*n*_(*ω*)} is drawn in (**a**) Figs 7 and (**b**) and 9. The solid lines denoted as SPP and SPPa are the *L*_||_ values corresponding to the *ε*_d_ − *ε*_m_ and *ε*_a_ − *ε*_m_ interface SPPs.

The values obtained for the realistic (lossy) case are shown in Fig. 8(a), together with reference values corresponding to SPPs on *ε*_d_ − *ε*_m_ (denoted as SPP) and *ε*_*a*_ − *ε*_m_ (denoted as SPPa) single interfaces. At lower frequencies, both TPM-2 and TPM-3 are seen to have *L*_||_ values between the two single-interface SPP limits. As *ω*_T_ is approached, the propagation length of TPM-3 drops which is a result of the reduced group velocity, cf. the reduced slope of TPM-3 in the vicinity of *ω*_T_ in Fig. 7(d). The *L*_||_ value of TPM-2 also drops above *ω*_T_, but here the reason is that it hybridizes with bulk modes in the superlattice and free space modes in the *ε*_*a*_ light cone and leaks away from the surface, cf. Fig. 7(b) where the thick (red) line denoted by 2 is seen to enter the *ε*_*a*_ lightcone once it enters the upper bulk band. Finally, TPM-1 is seen to have extremely low propagation lengths (below 100 nm) over the entire spectrum in which it is supported, which is the concequence of the vicinity of the SPP resonance at *ω*_spp_.

**Figure 9:**
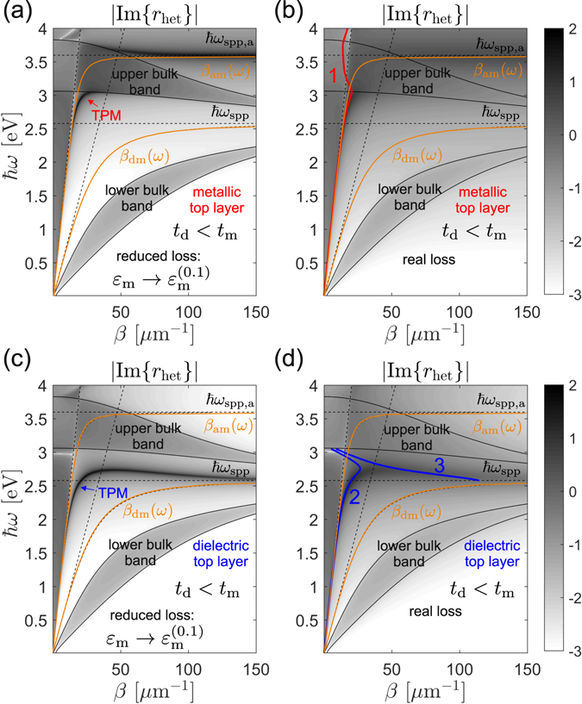
Maps of |Im{*r*_het_}| fully analogous to those drawn in Fig. 7, except that here the *t*_d_ < *t*_m_ example is considered.

Analogous *r*_het_ maps obtained for the *t*_d_ < *t*_m_ example are plotted in Fig. 9(a–d). As here *ω*_T_ > *ω*_spp_, homointerface-like TPMs appear only in the case of a dielectric top layer, as indicated by the TPM-2 and TPM-3 lines in Fig. 9(d). The shape of the TPM band indicated in Fig. 9(c) shows that the effect of replacing titanium dioxide by air as the capping medium blueshifts the homointerface TPM and deforms it so that its slope becomes zero around *β* ≈ 35 *μ*m^−1^ and negative for larger *β*. This implies that at frequencies slightly above *ω*_spp_ there are two TPM modes, which is confirmed by the numerically found TPM-2 and TPM-3 dispersion curves in Fig. 9(d). Here it should be noted that the TPM-3 curve represents −Re{*β*_3_}(*ω*), i.e. the actual dispersion curve lying in the *β* < 0 half-space is folded to the positive sided for a compact representation, see also refs^22,23^, for further discussion of this topic.

The TPM mode of the *t*_d_ < *t*_m_ superlattice with metallic top layer is a modified *ε*_*a*_ − *ε*_m_ single-interface SPP, as evident from its dispersion in Fig. 9(a) and (b), where it is seen to closely follow the *β*_am_(*ω*) curve, up until hybridizes with modes from the upper bulk band after which the pole shifts into the *ε*_*a*_ light cone and starts leaking out into both the superlattice and capping layer propagating modes. The propagation lengths of the TPM modes of the *t*_d_ < *t*_m_ example are shown in Fig. 8(b).

**Figure 10:**
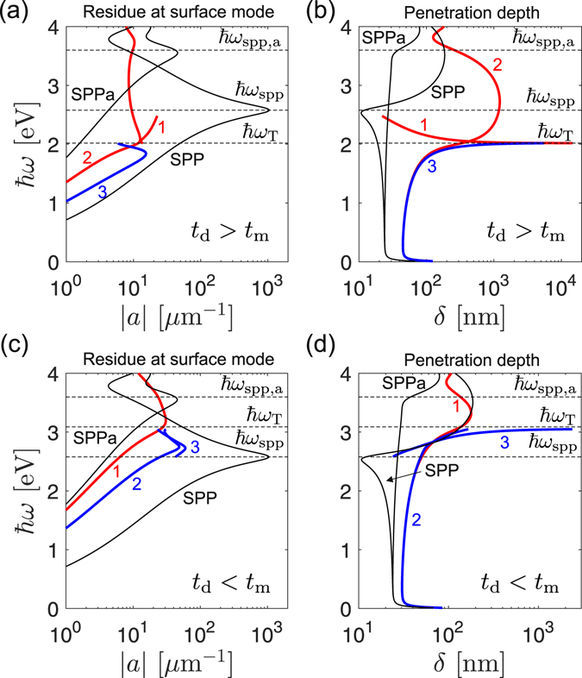
Left panels: residue at the TPM and SPP modes, drawn for the (**a**) *t*_d_ > *t*_m_ and (**c**) *t*_d_ < *t*_m_ examples, respectively. Righ panels: penetration depths into the superlattice for (**b**) *t*_d_ > *t*_m_ and (**d**) *t*_d_ < *t*_m_ examples, compared with the corresponding SPP penetration depth. SPP and SPPa stand for the *ε*_d_ − *ε*_m_ and *ε*_a_ − *ε*_m_ interface SPPs, respectively.

The resonance strength and confinement degree of heterointerface TPM modes for the considered two superlattice types is summarized in Fig. 10(a–d). Perhaps the most relevant case is that of the *t*_d_ > *t*_m_ supperlattice with a dielectric top layer supporting TPM-3 depicted in Fig. 10(a). Similarly as its homointerface counterpart, by the perturbation of which TPM-3 is formed, the resonance strength of this mode is seen to rapidly decrease as *ω*_T_ is approached while it becomes delocalized across the superlattice, as seen in Fig. 10(c).

### Summary and conclusion

This paper presents a systematic analysis of surface waves on two-phase semi-infinite metallodielectric superlattices. As their character is determined by the properties of the periodic arrangement of unit cells, in analogy with surface electronic states in crystals, these waves are termed Tamm plasmon modes.

It is shown that if the capping medium has the same permittivity as one of the superlattice constituents, a case referred to as the homointerface, TPM modes can exist only along the single-interface SPP dispersion curve and that there exists a critical frequency *ω*_T_ which represents the upper limit for the TPM existence in the dielectric- and the lower frequency limit in the metal-capped superlattice and is determined by the superlattice composition and relative layer thicknesses. This fact is particularly relevant if *ω*_T_ is below the SPP resonance frequency, which happens if the dielectric layers are thicker than the metal layers. Both the dielectric- and metal-capped TPMs become delocalized across the lattice when *ω*_T_ is approached, while the strength of the corresponding resonance, quantified by residues of the reflection coefficient at the associated poles, dies out.

The heterointerface, where the capping medium permittivity differs from that of the superlattice constituents and most relevant in practice as it includes the case of the superlattice exposed to air, is found to be exhibit a more complex behavior. We show that the heterointerface TPMs can be analyzed as resulting from either the capping-top layer interface, or an homointerface TPM located at the interface between the top layer and the rest of the superlattice. The homointerface-like TPMs, originating from the latter group, are found to also exhibit the critical behaviour around *ω*_T_.

In view of the significance of the effective medium approximation for the ongoing research on hyperbolic metamaterials, we have also compared its predictions against the exact theory. Somewhat surprisingly, we find that EMA accurately describes several important aspects of homointerface TPMs with arbitrary layer thicknesses, including the critical behaviour, but that it fails entirely in the heterointerface case.

These results are important for engineering the optical properties of semi-infinite metallodielectric superlattices which have recently been receiving strong attention in connection with enhanced spontaneous emission into the bulk superlattice modes. The analysis reported here should help quantify the individual contributions of the bulk and TPM modes in the total optical density of states at the interface and thus tailor the amount of emitted light that propagates through the superlattice or stays tied at the interface, respectively.
