# Publisher Correction: Tamm plasmon modes on semi-infinite metallodielectric superlattices

**DOI:** 10.1038/s41598-018-22652-8

**Published:** 2018-03-08

**Authors:** Goran Isić, Slobodan Vuković, Zoran Jakšić, Milivoj Belić

**Affiliations:** 1 Institute of Physics Belgrade, Center for Solid State Physics and New Materials, University of Belgrade, Belgrade, 11080 Serbia; 2 Institute of Chemistry, Technology and Metallurgy, Center Of Microelectronic Technologies, University of Belgrade, Belgrade, 11000 Serbia; 3 Texas A&M University at Qatar, Doha, P.O. Box 23874 Qatar

Correction to: *Scientific Reports* 10.1038/s41598-017-03497-z, published online 16 June 2017

The original version of this Article contained a typographical error in the spelling of the author Zoran Jakšić, which was incorrectly given as Zoran Jašić. This has now been corrected in the PDF and HTML versions of the Article.

